# Validity and diagnostic accuracy of the Luganda version of the 9-item and 2-item Patient Health Questionnaire for detecting major depressive disorder in rural Uganda

**DOI:** 10.1017/gmh.2016.14

**Published:** 2016-06-20

**Authors:** J. E. M. Nakku, S. D. Rathod, D. Kizza, E. Breuer, K. Mutyaba, E. C. Baron, J. Ssebunnya, F. Kigozi

**Affiliations:** 1 Butabika Hospital/Makerere University College of Health Sciences, Kampala, Uganda; 2 Department of Population Health, London School of Hygiene and Tropical Medicine, London, UK; 3 Department of Psychiatry and Mental Health, Alan J Flisher Centre for Public Mental Health, University of Cape Town, South Africa

**Keywords:** Depression screening, PHQ-9, validation

## Abstract

**Background.:**

The prevalence of depression in rural Ugandan communities is high and yet detection and treatment of depression in the primary care setting is suboptimal. Short valid depression screening measures may improve detection of depression. We describe the validation of the Luganda translated nine- and two-item Patient Health Questionnaires (PHQ-9 and PHQ-2) as screening tools for depression in two rural primary care facilities in Eastern Uganda.

**Methods.:**

A total of 1407 adult respondents were screened consecutively using the nine-item Luganda PHQ. Of these 212 were randomly selected to respond to the Mini International Neuropsychiatric Interview diagnostic questionnaire. Descriptive statistics for respondents’ demographic characteristics and PHQ scores were generated. The sensitivity, specificity and positive predictive values (PPVs), and area under the ROC curve were determined for both the PHQ-9 and PHQ-2.

**Results.:**

The optimum trade-off between sensitivity and PPV was at a cut-off of ≧5. The weighted area under the receiver Operating Characteristic curve was 0.74 (95% CI 0.60–0.89) and 0.68 (95% CI 0.54–0.82) for PHQ-9 and PHQ-2, respectively.

**Conclusion.:**

The Luganda translation of the PHQ-9 was found to be modestly useful in detecting depression. The PHQ-9 performed only slightly better than the PHQ-2 in this rural Ugandan Primary care setting. Future research could improve on diagnostic accuracy by considering the idioms of distress among Luganda speakers, and revising the PHQ-9 accordingly. The usefulness of the PHQ-2 in this rural population should be viewed with caution.

## Background

Depression is common, chronic and costly for both sufferers and health care systems. It is estimated to be a leading cause of disability worldwide but data on population rates of depression in developing countries remain sparse (Whiteford *et al*. 2013). Two surveys conducted among adults living in rural areas of Uganda found the point prevalence of major depression to be between 17% and 24% (Bolton *et al*. 2003; Ovuga *et al*. 2005).

Despite the high burden and substantial impact of depression, detection and treatment in the primary care setting are suboptimal. In high-income countries, primary care physicians fail to recognize 30%–50% of depressed patients (Simon & VonKorff, 1995). This diagnosis gap is estimated to be even more substantial in low-income countries (Kohn *et al*. 2004; Wang *et al*. 2007).

Short valid depression screening measures such as the nine-item Patient Health Questionnaire (PHQ-9) (Kroenke *et al*. 2001) may help primary care health workers identify patients with depressive symptoms Pignone *et al*. (2002) in their systematic review of randomized controlled studies of depression screening in primary care settings showed that use of screening instruments led to increased recognition of depression in primary care in the USA.

The PHQ-9 has been validated in a variety of settings worldwide (Wittkampf *et al*. 2007; Manea *et al*. 2012). In sub-Saharan Africa, validation studies have been conducted among university students in Nigeria (Adewuya *et al*. 2006) and postnatal women in Ghana (Weobong *et al*. 2009). Studies have also been conducted among people living with HIV in Kenya (Monahan *et al*. 2009), Cameroon (Pence *et al*. 2012) and in Uganda (Akena *et al*. 2013), where they found an AUROC of 0.96. The PHQ-9 and two-item Patient Health Questionnaire (PHQ-2) has been recently validated among general health facility attendees in Ethiopia (Hanlon *et al*. 2015) and South Africa (Bhana *et al*. 2015). However, no such study has yet been conducted in Uganda. Understanding the diagnostic accuracy and validity of the PHQ-9 is essential if the PHQ-9 is going to be used as a screening tool to increase detection of depression by non-mental health professionals, improve opportunity for mental health care and reduce the depression treatment gap in Uganda.

This study therefore aims to confirm the validity and diagnostic accuracy of the Luganda PHQ-9, and of a shortened version of the PHQ-9, the PHQ-2, in detecting depression among an adult population attending primary care clinics in rural Uganda. It also aims to examine the internal structure of the translated tool.

## Methods

### Study setting

Kamuli District was selected for the integration of mental health services for depression, alcohol use disorder, psychosis and epilepsy into primary health care sector as part of the Programme for Improving Mental Health Care (PRIME) (Lund *et al*. 2012) in collaboration with the Uganda Ministry of Health (Kigozi *et al*. 2016). Kamuli District has a population of 490 000, of whom 96% reside in rural areas (Uganda Bureau of Statistics, 2016). This population is served by 41 primary health care centres and two hospital outpatient departments. Primary care centres in Kamuli provide first level outpatient health care, including treatment of acute infections, minor accidents, maternal and child health services and basic mental health assessments treatment and referral. Most patients with depression or suicide will come to the health centre or outpatient department of a hospital with somatic complaints or physical consequences of a suicide attempt, which may or may not trigger a psychiatric assessment, treatment or referral. Up to 100 patients may be seen a day at the health centres, all conditions put together. The district hospital's outpatient department operates like a primary care facility as a first point of contact for many patients in the hospital's catchment area. The district hospital outpatient department sees two to three times the number of patients seen at the lower health centres.

### Participants

Research assistants recruited patients attending one primary health and one hospital outpatient department into the study between October and December 2014. Participants were eligible for the study if they were at least 18 years of age, fluent in Luganda, were attending the clinic for primary care and were available for a 30 min interview. Participants were excluded if they required acute medical care. Participants were recruited into a convenience sample, dictated by the availability of the research assistants to recruit the next patient who arrived to the clinic waiting room.

### Study procedures

Recruitment and screening was done by two research assistants who were general nurses working in Kamuli. They had a 2-day training in the PHQ-9 administration and spoke both English and Luganda. After assessing the patient for inclusion and exclusion criteria, the research assistant explained the study's purpose and procedures in the local language to ensure that even those participants with low literacy understood. Signed (or thumb printed) informed consent was obtained from patients who consented to participate in the study.

In a private examination room, the research assistant collected demographic information from the participant, administered the Luganda version of the PHQ-9 on paper and calculated the total screening score. The research assistant then selected a sealed envelope, inside of which contained pre-printed set of instructions. The instructions randomly allocated participants to either the Mini International Neuropsychiatric Interview (MINI) or no MINI interview, so that a subsample of 200 participants would be referred to the diagnostic interview. An example of such an instruction is: ‘is the total score X? If yes, send respondent to MINI interview. If no, select the next person who scores *X* to go to the MINI interview.’ The screened but un-referred participants were immediately returned to the waiting room to be managed by the clinic staff.

The diagnostic interview was conducted within minutes of the PHQ-9 interview in a room adjacent to the outpatient department. Both the participant and the diagnostician were blind to the participant's PHQ-9 screening score. The diagnosticians were mental health practitioners (one psychiatric clinical officer and one psychiatric nurse with a diploma in clinical medicine and nursing, respectively) who were fluent in Luganda. The diagnosticians completed a 2-day training in the use of the depression module of the MINI diagnostic interview.

After the diagnostic interview was completed the research assistant brought the participants back to the waiting room. However, patients who responded positively to the suicide question either on PHQ-9 or in the diagnostic interview were taken directly to a primary care health worker for further assessment and management by the primary care worker who was informed about the need to assess and manage for suicidality.

### Assessments

#### Patient health questionnaire (PHQ-9)

The PHQ-9 is the nine-item depression scale of the Patient Health Questionnaire (Spitzer *et al*. 1994). The nine items of the PHQ-9 are based directly on the nine diagnostic criteria for major depressive disorder in the DSM-IV (American Psychiatric Association, 1994). Each item is scored on a likert scale with symptoms rated as 0 (not at all), 1 (several days), 2 (more than half the days) and 3 (nearly every day).The PHQ-2 is a two-item version of the PHQ-9. It is scored in the same manner as the PHQ-9. It is advantageous for use by general clinicians in busy health care facilities.

The English version of the PHQ-9 was translated into Luganda by two bilingual translators. This was followed by evaluation of the translated tools by two bilingual clinical Psychologists who were familiar with the constructs in the tool in order to gain consensus on the translation. The consensus version of the questionnaire was then piloted in a small sample of 30 respondents in a small health facility that was not the study facility before the start of the study. This pilot served to ensure linguistic and idiomatic equivalence and to identify any challenges respondents might have with the translation. Any unclear Luganda terms were modified to include terms that are more commonly used and understandable by the participants in order to produce a final PHQ-9 Luganda translation. This modification of Luganda terms did not fundamentally change the items of the PHQ-9. The Luganda translation of the PHQ-9 is available in the additional files of this manuscript.

#### Mini-International Neuropsychiatric Inter (MINI) - Depression module

The depression module of the MINI was used as the gold standard. The MINI is a structured diagnostic interview designed for research and clinical practice in psychiatric and primary care settings. The depression module of the MINI 5.0.0 (Sheehan *et al.*, 1998) is used to diagnose current major depressive disorder. The MINI depression module was translated into Luganda using the same process as for the PHQ-9 described above. This process included the initial translation of the English version, building consensus by bilingual mental health experts, piloting and modification of difficult terms to produce a final version. There was no change in the core items of the MINI questionnaire.

### Sample size

Given constraints on the availability of diagnosticians, a validation sample size of 200 diagnostic interviews was fixed. The anticipated distribution of PHQ-9 scores came from a study previously conducted with adult attendees in public clinics in Kamuli in 2013, which was characterized by a right skew. Accordingly, in order to refer 200 participants with balanced distribution of PHQ-9 scores, 1300 participants needed to be screened with 7% of participant with low (0–2) scores, 11% of participants with medium (3–7) scores and 100% of participants with high (≥8) scores being referred to complete the diagnostic interview. A balance of scores in the analysis dataset would allow for more precise estimates of the sensitivity and specificity of the PHQ-9 and PHQ-2 at higher cut points.

### Statistical methods

Descriptive statistics were used to describe the demographic characteristics of the participants, their PHQ-9 scores and the distribution of PHQ-9 and PHQ-2 scores in the sample. The factor structure of the Luganda PHQ-9 was examined using principle components analysis with varimax rotation, a Scree plot and the Kaiser test. The internal consistency of the Luganda PHQ-9 was calculated using Cronbach's alpha. The correlation of each item of the PHQ-9 was assessed against the sum of the other eight items (the item-rest correlation).

Diagnostic validity of the PHQ-9 and the PHQ-2 was then evaluated using the area under the receiver operating curve (AUROC). The area under the receiver operating characteristic curve (AUROC) of the PHQ-9 and the PHQ-2 were estimated using the Somers’ D with Harrell's c statistic (Newson, 2002), which allowed for estimation of the AUROC with probability weights. The 95% confidence intervals for the weighted AUROCs were based on bootstrap distribution of the AUROC estimation procedures, using 5000 repetitions (Alonzo & Pepe, 2005).

### Ethics

The study procedures were approved by the human subject protections boards at Makerere University (Kampala, Uganda) and the University of Cape Town (South Africa).

## Results

We obtained informed consent from 1415 patients of which a total of 1407 were screened on the PHQ-9 from both the health centre and the district hospital outpatient department. Eight participants could not complete the PHQ-9 because they were in a hurry.

**Fig. 1.**
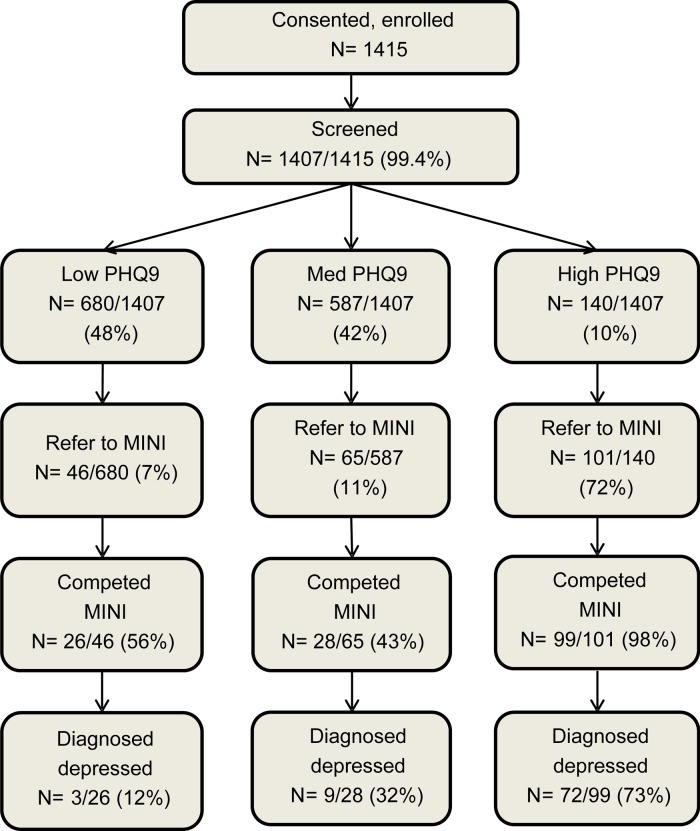
Participant selection and screening for PHQ-9 validation.

Nearly half (48.3%) of participants had a low PHQ-9 score (0–2), a similar proportion (41.7%) had a medium score (5–7) and 10.0% had a high score (8 and above). A total of 212 (15.1% of 1407) participants were referred to the MINI diagnostic interview, of whom 153 (72.2% of 212) completed the diagnostic interview. The rest withdrew their participation due to time constraints. The flow diagram of participants’ screening scores and referral to the MINI diagnostic is shown in Fig. 1.

### Demographic and clinical characteristics of the sample

In the total sample, the participants’ mean age was 32.6 years (s.d. 12.6, range 18–82) and 72.3% were female. Of the female respondents, 17.5% were in the perinatal period (defined as being pregnant or having delivered in the past 12 months). Nearly half (48.0%) of participants had completed primary school, while another 31.2% completed secondary school and 7.2% had completed some higher education. Many participants (43.6%) were unemployed, while 42.7% were engaged in paid or self-employment. Another 9.1% of participants were students.

The median PHQ-9 score was 3 [interquartile range (IQR) 1–5, range 0–25] and the median PHQ-2 score was 1 (IQR 0–2, range 0–6).

Among the 153 participants who completed the MINI diagnostic interview, 84 (57%) were found to have major depressive disorder. Seven per cent (103 of 1407) of participants reported having thoughts about suicide or self-harm on PHQ-9 item no. 9.

### Psychometric properties of the PHQ-9

Two analysis techniques showed two different factor structures of the PHQ-9. The Scree plot after varimax rotation of Principal Component Analysis suggested a two-factor solution (total variance explained = 43%) while the Kaiser criteria suggested a three-factor solution (total variance explained = 57%). With three factors, PHQ items no. 2, 3 and 5 load onto factor 1, PHQ items no. 6, 8 and 9 onto factor 2 and PHQ items no. 1, 4 and 7 onto factor 3. Evidence of a multi-factor solution precludes a coherent interpretation of the Cronbach's alpha for the internal consistency of the PHQ-9, which was 0.68. The item-rest correlations were all below 0.5: item no. 6 had the highest correlation (0.48), while the lowest correlations were for item no. 1 (0.19) and item no. 4 (0.23).

### Validation of the PHQ-9 and PHQ-2

**Fig. 2.**
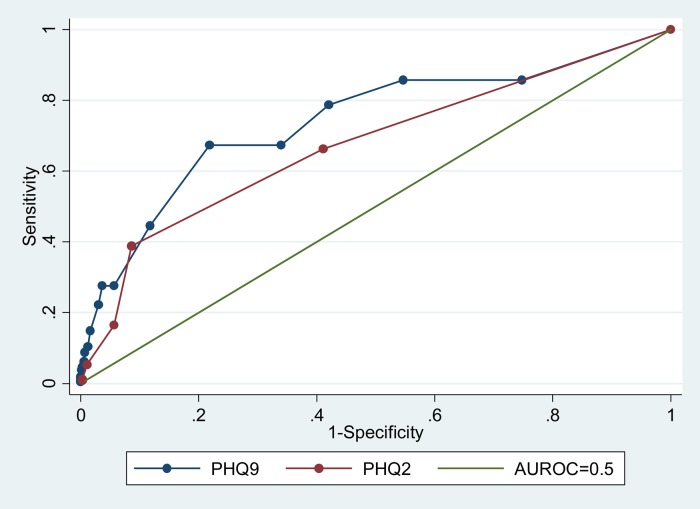
Receiver operating curve for PHQ-9 and PHQ-2 for adults in primary health care facilities in Kamuli, Uganda, 2014.

The weighted area under the receiver Operating Characteristic curve for the PHQ-9 was 0.74 (95% CI 0.60–0.89) and for the PHQ-2 was 0.68 (95% CI 0.54–0.82) (Fig. 2).

Table 1 below shows the specificity, sensitivity and positive predictive values (PPVs) of the PHQ-9 and PHQ-2 at different cut-off scores. In this study, the optimum trade-off between sensitivity and PPV was at a cut-off of ≧5. It means therefore that at this cut-off, 67% of people with depression are screen positive, 78% of people without depression are screen negative, and 52% of screen positive people have depression. On the PHQ-2, at a cut-of ≧1, 66% of people with depression are screen positive, 59% of people without depression are screen negative and 36% of people who are screen positive have depression.

**Table 1.** Diagnostic characteristics of the PHQ-9 and PHQ-2 for major depression among adults in primary care facilities in Kamuli District, Uganda, 2014

	PHQ-9			PHQ-2	
	SENS	SPECI	PPV	SENS	SPECI
≥0	100.0	0.0	26.2	100.0	0.0
≥1	85.8	25.2	29.0	66.2	58.9
≥2	85.8	45.4	35.8	38.9	91.4
≥3	78.7	57.9	40.0	16.5	94.3
≥4	67.4	66.0	41.3	5.4	98.9
≥5	67.4	78.1	52.3	1.1	99.7
≥6	44.6	88.2	57.4	0.8	99.7
≥7	27.6	94.3	63.3		
≥8	27.6	96.3	72.7		
≥9	22.2	97.0	72.5		
≥10	14.9	98.4	76.5		
≥11	10.3	98.8	75.0		
≥12	8.8	99.3	82.1		

## Discussion

This paper reports the psychometric properties and diagnostic accuracy of the Luganda version of the nine-item and two-item PHQ in a primary health care setting in rural Uganda. Our results showed: (1) depression is a multi-faceted construct in this setting; and (2) that the PHQ-9 has diagnostic utility in this setting; (3) that the PHQ-2 did not perform well in this rural population. However, a trade-off between high sensitivity and low PPV must be noted.

The diagnostic characteristics of the PHQ-9 (AUROC = 0.74) and the PHQ-2 (AUROC = 0.68) indicate that the PHQ-9 performance in the rural population studied was modest. However, it could still be used to screen for depression in primary care facilities in rural Uganda. The PHQ-9 performed only slightly better than the PHQ-2. The area under the receiver operating characteristic curve in this study for both PHQ-9 and PHQ-2 was smaller than that found in similar settings in Ethiopia (PHQ-9, AUROC = 0.85 and PHQ-2, AUROC = 0.78) where the PHQ was compared with a psychiatric nurse administered MINI (Hanlon *et al*. 2015) and in South Africa (PHQ-9, AUROC = 0.85 and PHQ-2, AUROC = 0.76) where the PHQ was compared with clinical psychologist administered structured clinical interview for DSM-IV (Bhana *et al*. 2015). The choice of MINI as a gold standard, which has not been validated in Uganda, could have led to the modest validity of the Luganda PHQ-9 compared with that found in other PHQ-9 validation studies in sub-Saharan Africa. Also the local idioms of distress could have been different from the items in the PHQ-9 leading to lower validity than would have been expected. For example Item no. 1 (*little interest or pleasure in doing things*) is often not understood and is difficult to translate to Luganda for one to understand. Item no. 2 (*feeling down, depressed or hopeless*) is often expressed as one ‘having thoughts’ (Okello & Ekblad, 2006), which is a cognitive construct rather than feeling sad which is an emotional construct. This item therefore may not be understood the same way or responded to appropriately in its current form on the PHQ-9.

We suggest a potential cut-off score of ≧5 although we note that at this there is a significant trade-off between sensitivity and low PPV. The cut-off is similar to that obtained in Nigeria among University students where the PHQ-9 was validated against the MINI (Adewuya *et al*. 2006). This cut-off is, however, lower than what was recommend by other studies done both in sub-Saharan Africa (Omoro *et al*. 2006) and globally (Manea *et al*. 2012). In a previous meta-analysis of 18 studies, Manea *et al*. (2012) found that the PHQ-9 performed well at cut-off scores between 8 and 11, though only one of these studies was from sub-Saharan Africa. Therefore, as proposed by Patel *et al*. (2008), we recommend that the choice of an optimal cut-off score based on the best balance between sensitivity and PPV may need to be tailored to individual settings and purpose. This is particularly important for routine use in low-income countries where the burden on healthcare staff is high (Kagee *et al*. 2013) and referral of negative cases may put additional strain on an already struggling health system.

The presence of two to three factors on the factor analysis provides evidence that depression is a multi-faceted construct in this setting. Several other studies of the psychometric properties of the PHQ-9 found the somatic and non-somatic symptoms of depression loading onto different factors (Richardson & Richards, 2008; Kalpakjian *et al*. 2009; Chilcot *et al*. 2013; Zhong *et al*. 2014; Petersen *et al*. 2015). Interpretation of these findings is further complicated by the relatively high prevalence of somatic symptoms reported by adults recruited from primary care facilities, and those symptoms’ conflation with depressive symptoms. While clinicians must take care to confirm that a high screening score is not an artefact of some other underlying disorder, they must also be sensitized to the non-specific manifestation of depressive symptoms and the likelihood of mis-diagnosis or under-diagnosis. In particular item no. 1 (*little interest or pleasure in doing things*) and item no. 4 (*feeling tired or having little energy*) of the PHQ-9 were poorly correlated with the total PHQ-9 score and both loaded on to the same factor. These items are strong candidates for further evaluation as to whether they are able to distinguish between depressive and non-depressive symptoms in an outpatient population and whether their translation captures the correct idiom of distress.

This is the first study to validate the PHQ-9 and PHQ-2 in a Ugandan primary health care population. A previous validation study had been done in a highly selected HIV population (Akena *et al*. 2013). However, the strengths notwithstanding, there were some limitations. Firstly, the findings from this study may not be generalizable to non-Luganda speakers or to populations outside health facilities. Secondly, there was substantial loss to follow up among participants who were referred for the MINI diagnostic assessment. Both the estimates of prevalence and the AUROC could be biased by this loss to follow up. Lastly, because the MINI administration was dependent on the PHQ-9 score, the MINI interview had to be conducted after the PHQ-9. It is possible that the PHQ-9 primed participants into being more open to discuss their depressive symptoms during the diagnostic interview, which would bias the AUROC estimates downward. However, given that mental health services were not yet available in this district, the participants remained ‘blind’ to their depression status, which is a source of bias common in other validation studies of depression screening tools (Thombs *et al*. 2011).

## Conclusion

The Luganda translation of the PHQ-9 was found to have modest validity in detecting depression. In clinics where staff have limited time, one would hope that the PHQ-2 could be a useful alternative. However, in this study, the PHQ-9 performed slightly better than the PHQ-2. If one chose to screen with the PHQ-2 therefore, one would need to follow it up with a more detailed tool in order to accurately discriminate between those with depression and those without. Future research could improve on diagnostic accuracy by considering the factor structure of the Luganda PHQ-9, considering the idioms of distress among Luganda speakers, and revising the PHQ-9 accordingly.

## Supplementary material

For supplementary material accompanying this paper visit http://dx.doi.org/10.1017/gmh.2016.14. click here to view supplementary material

## References

[ref1] Adewuya AO, Ola BA, Afolabi OO (2006). Validity of the patient health questionnaire (PHQ-9) as a screening tool for depression amongst Nigerian university students. Journal of Affective Disorders 96, 89–93. 16857265 10.1016/j.jad.2006.05.021

[ref2] Akena D, Joska J, Obuku EA, Stein DJ (2013). Sensitivity and specificity of clinician administered screening instruments in detecting depression among HIV-positive individuals in Uganda. AIDS Care 25, 1245–1252. 23398282 10.1080/09540121.2013.764385

[ref3] Alonzo TA, Pepe MS (2005). Assessing accuracy of a continuous screening test in the presence of verification bias. Journal of the Royal Statistical Society: Series C (Applied Statistics) 54, 173–190.

[ref4] American Psychiatric Association (1994). Diagnostic and Statistical Manual of Mental Disorders, 4th edn, (DSM-IV). APA: Washington, DC.

[ref5] Bhana A, Rathod SD, Selohilwe O, Kathree T, Petersen I (2015). The validity of the Patient Health Questionnaire for screening depression in chronic care patients in primary health care in South Africa. BMC Psychiatry 15, 118. 26001915 10.1186/s12888-015-0503-0 PMC4446842

[ref6] Bolton P, Bass J, Neugebauer R, Verdeli H, Clougherty KF, Wickramaratne P, Speelman L, Ndogoni L, Weissman M (2003). Group interpersonal psychotherapy for depression in rural Uganda. JAMA 289, 3117–3124. 12813117 10.1001/jama.289.23.3117

[ref7] Chilcot J, Rayner L, Lee W, Price A, Goodwin L, Monroe B, Sykes N, Hansford P, Hotopf M (2013). The factor structure of the PHQ-9 in palliative care. Journal of Psychosomatic Research 75, 60–64. 23751240 10.1016/j.jpsychores.2012.12.012

[ref8] Hanlon C, Medhin G, Selamu M, Breuer E, Worku B, Hailemariam M, Lund C, Prince M, Fekadu A (2015). Validity of brief screening questionnaires to detect depression in primary care in Ethiopia. Journal of Affective Disorders 186, 32–39. 26226431 10.1016/j.jad.2015.07.015

[ref9] Kagee A, Tsai AC, Lund C, Tomlinson M (2013). Screening for common mental disorders in low resource settings: reasons for caution and a way forward. International Health 5, 11–14. 23580905 10.1093/inthealth/ihs004 PMC3619733

[ref10] Kalpakjian CZ, Toussaint LL, Albright KJ, Bombardier CH, Krause JK, Tate DG (2009). Patient Health Questionnaire-9 in spinal cord injury: an examination of factor structure as related to gender. Journal of Spinal Cord Medicine 32, 147. 19569462 10.1080/10790268.2009.11760766 PMC2678286

[ref11] Kigozi FN, Kizza D, Nakku J, Ssebunnya J, Ndyanabangi S, Nakiganda B, Lund C, Patel V (2016). Development of a district mental healthcare plan in Uganda. British Journal of Psychiatry 208, s40–s46. 26447171 10.1192/bjp.bp.114.153742 PMC4698555

[ref12] Kohn R, Saxena S, Levav I, Saraceno B (2004). The treatment gap in mental health care. Bulletin of the World Health Organization 82, 858–866. 15640922 PMC2623050

[ref13] Kroenke K, Spitzer RL, Williams JB (2001). The PHQ-9. Validity of a brief depression severity measure. Journal of General Internal Medicine 16, 606–613. 11556941 10.1046/j.1525-1497.2001.016009606.x PMC1495268

[ref14] Lund C, Tomlinson M, de Silva M, Fekadu A, Shidhaye R, Jordans M, Petersen I, Bhana A, Kigozi F, Prince M, Thornicroft G, Hanlon C, Kakuma R, McDaid D, Saxena S, Chisholm D, Raja S, Kippen-Wood S, Honikman S, Fairall L, Patel V (2012). PRIME: a programme to reduce the treatment gap for mental disorders in five low- and middle-income countries. PLoS Medicine 9, e1001359. 23300387 10.1371/journal.pmed.1001359 PMC3531506

[ref15] Manea L, Gilbody S, McMillan D (2012). Optimal cut-off score for diagnosing depression with the Patient Health Questionnaire (PHQ-9): a meta-analysis. CMAJ 184, E191–E196. 22184363 10.1503/cmaj.110829 PMC3281183

[ref16] Monahan PO, Shacham E, Reece M, Kroenke K, Ong'or WO, Omollo O, Yebei VN, Ojwang C (2009). Validity/reliability of PHQ-9 and PHQ-2 depression scales among adults living with HIV/AIDS in western Kenya. Journal of General Internal Medicine 24, 189–197. 19031037 10.1007/s11606-008-0846-z PMC2629000

[ref33] Newson R (2002). Parameters behind “nonparametric” statistics: Kendall's tau, Somers' D and median differences. Stata Journal 2, 45–64.

[ref34] Okello ES, Ekblad S (2006). Lay concepts of depression among the Baganda of Uganda: a pilot study. Transcultural Psychiatry 43, 287–313. 16893877 10.1177/1363461506064871

[ref18] Omoro SA, Fann JR, Weymuller EA, Macharia IM, Yueh B (2006). Swahili translation and validation of the Patient Health Questionnaire-9 depression scale in the Kenyan head and neck cancer patient population. International Journal of Psychiatry in Medicine 36, 367–381. 17236703 10.2190/8W7Y-0TPM-JVGV-QW6M

[ref19] Ovuga E, Boardman J, Wasserman D (2005). The prevalence of depression in two districts of Uganda. Social Psychiatry and Psychiatric Epidemiology 40, 439–445. 16003593 10.1007/s00127-005-0915-0

[ref20] Patel V, Araya R, Chowdhary N, King M, Kirkwood B, Nayak S, Simon G, Weiss HA (2008). Detecting common mental disorders in primary care in India: a comparison of five screening questionnaires. Psychological Medicine 38, 221–228. 18047768 10.1017/S0033291707002334 PMC4959557

[ref21] Pence BW, Gaynes BN, Atashili J, O'Donnell JK, Tayong G, Kats D, Whetten R, Whetten K, Njamnshi AK, Ndumbe PM (2012). Validity of an interviewer-administered patient health questionnaire-9 to screen for depression in HIV-infected patients in Cameroon. Journal of Affective Disorders 143, 208–213. 22840467 10.1016/j.jad.2012.05.056 PMC3500577

[ref22] Petersen JJ, Paulitsch MA, Hartig J, Mergenthal K, Gerlach FM, Gensichen J (2015). Factor structure and measurement invariance of the Patient Health Questionnaire-9 for female and male primary care patients with major depression in Germany. Journal of Affective Disorders 170, 138–142. 25240840 10.1016/j.jad.2014.08.053

[ref23] Pignone MP, Gaynes BN, Rushton JL, Burchell CM, Orleans CT, Mulrow CD, Lohr KN (2002). Screening for depression in adults: a summary of the evidence for the U.S. Preventive Services Task Force. Annals of Internal Medicine 136, 765–776. 12020146 10.7326/0003-4819-136-10-200205210-00013

[ref24] Richardson EJ, Richards JS (2008). Factor structure of the PHQ-9 screen for depression across time since injury among persons with spinal cord injury. Rehabilitation Psychology 53, 243.

[ref35] Sheehan DV, Lecrubier Y, Sheehan KH, Amorim P, Janavs J, Weiller E, Hergueta T, Baker R, Dunbar GC (1998). The Mini-International Neuropsychiatric Interview (MINI): the development and validation of a structured diagnostic psychiatric interview for DSM-IV and ICD-10. Journal of Clinical Psychiatry 59 (Suppl 20), 22–33. 9881538

[ref25] Simon GE, VonKorff M (1995). Recognition, management, and outcomes of depression in primary care. Archives of Family Medicine 4, 99. 7842160 10.1001/archfami.4.2.99

[ref26] Spitzer RL, Williams JB, Kroenke K, Linzer M, Verloin deGruy F, Hahn SR, Brody D, Johnson JG (1994). Utility of a new procedure for diagnosing mental disorders in primary care: the PRIME-MD 1000 study. JAMA 272, 1749–1756. 7966923

[ref36] Thombs BD, Arthurs E, El-Baalbaki G, Meijer A, Ziegelstein RC, Steele RJ (2011). Risk of bias from inclusion of patients who already have diagnosis of or are undergoing treatment for depression in diagnostic accuracy studies of screening tools for depression: systematic review. BMJ 343, d4825. 21852353 10.1136/bmj.d4825 PMC3191850

[ref27] Uganda Bureau of Statistics (2016). National Population and Housing Census Final Report 2014. http://library.health.go.ug/publications/leadership-and-governance-monitoring-and-evaluation/population/national-population-an-0.

[ref28] Wang PS, guilar-Gaxiola S, Alonso J, Angermeyer MC, Borges G, Bromet EJ, Bruffaerts R, de GG, de GR, Gureje O, Haro JM, Karam EG, Kessler RC, Kovess V, Lane MC, Lee S, Levinson D, Ono Y, Petukhova M, Posada-Villa J, Seedat S, Wells JE (2007). Use of mental health services for anxiety, mood, and substance disorders in 17 countries in the WHO world mental health surveys. Lancet 370, 841–850. 17826169 10.1016/S0140-6736(07)61414-7 PMC2847360

[ref29] Weobong B, Akpalu B, Doku V, Owusu-Agyei S, Hurt L, Kirkwood B, Prince M (2009). The comparative validity of screening scales for postnatal common mental disorder in Kintampo, Ghana. Journal of Affective Disorders 113, 109–117. 18614241 10.1016/j.jad.2008.05.009

[ref30] Whiteford HA, Degenhardt L, Rehm J, Baxter AJ, Ferrari AJ, Erskine HE, Charlson FJ, Norman RE, Flaxman AD, Johns N (2013). Global burden of disease attributable to mental and substance use disorders: findings from the Global Burden of Disease Study 2010. Lancet 382, 1575–1586. 23993280 10.1016/S0140-6736(13)61611-6

[ref31] Wittkampf KA, Naeije L, Schene AH, Huyser J, van Weert HC (2007). Diagnostic accuracy of the mood module of the Patient Health Questionnaire: a systematic review. General Hospital Psychiatry 29, 388–395. 17888804 10.1016/j.genhosppsych.2007.06.004

[ref32] Zhong Q, Gelaye B, Rondon M, Sánchez SE, García PJ, Sánchez E, Barrios YV, Simon GE, Henderson DC, Cripe SM (2014). Comparative performance of patient health questionnaire-9 and Edinburgh Postnatal Depression Scale for screening antepartum depression. Journal of Affective Disorders 162, 1–7. 24766996 10.1016/j.jad.2014.03.028 PMC4040145

