# Supplementary material for: Validity and diagnostic accuracy of the Luganda version of the 9-item and 2-item Patient Health Questionnaire for detecting major depressive disorder in rural Uganda
Source: Glob Ment Health (Camb). 2016 Jun 20;3:e20. doi: 10.1017/gmh.2016.14 (PMC5314749; doi:10.1017/gmh.2016.14)
Supplement: Supplementary file 1 [file S2054425116000145sup001.docx]

| PHQ 9 QUESTIONNAIRE- LUGANDA VERSION | | | | |
| --- | --- | --- | --- | --- |
| Ngenda kukunyonyola engeri ezimu abantu abomu gyw bewuliramu, era kubuzze oba nga ekimu kukyo kyali kikutuseeko mu wiki ebiri eziyise.  Oluvanyuma lwe byo ebibuzo ebyo, nja kubuza oba obadde otawanyizibwa ebizibu ebyo byebimu okumala akaseera aka sabiti ebiiri oba okusingawo mumwaka oguyise.  Muwiiki ebiri eziyise, mirundi emeeka bino ebizibu wamanga gye bibadde okutawanyamu? | | | | |
| 1 | Obutanyumirwa oba okusanyusibwa mukukola ebintu eby’enjawulo | Nedda | 0 |  |
|  |  | Ennaku ezimu | 1 |  |
|  |  | Enaku ezisinga obungi | 2 |  |
|  |  | Kumpi buli lunaku | 3 |  |
| 2 | Okubeera omunakuwavu oba omwenyamivu oba ng’owulira obulamu tebukyalina makulu | Nedda | 0 |  |
|  |  | Enaku ezimu | 1 |  |
|  |  | Enaku ezisinga obungi | 2 |  |
|  |  | Kumpi buli lunaku | 3 |  |
| 3 | Obuzibu mu kufuna otulo, oba mu kwebaka ekimala; oba okwebaka nnyo | Nedda | 0 |  |
|  |  | Enaku ezimu | 1 |  |
|  |  | Enaku ezisinga obungi | 2 |  |
|  |  | Kumpi buli lunaku | 3 |  |
| 4 | Okuwulira obukowu oba okubeera n’amanyi amatono | Nedda | 0 |  |
|  |  | Enaku ezimu | 1 |  |
|  |  | Enaku ezisinga obungi | 2 |  |
|  |  | Kumpi buli lunaku | 3 |  |
| 5 | Obutawomerwa/obutayagala byakulya oba okwagala okulya ennyo | Nedda | 0 |  |
|  |  | Enaku ezimu | 1 |  |
|  |  | Enaku ezisinga obungi | 2 |  |
|  |  | Kumpi buli lunaku | 3 |  |
| 6 | Okwekyawa mubulamu bwo - oba okweraba ng’atakyali wamugaso munsi; nga olaba olemeredwa mubyonna, nabafamile tebakyakulabawo. | Nedda | 0 |  |
|  |  | Enaku ezimu | 1 |  |
|  |  | Enaku ezisinga obungi | 2 |  |
|  |  | Kumpi buli lunaku | 3 |  |
| 7 | Obuzibu mukusaayo omwoyo mu by’okola okugeza nga okusoma e mpapula z’amawulire oba okulaba T.V. | Nedda | 0 |  |
|  |  | Enaku ezimu | 1 |  |
|  |  | Enaku ezisinga obungi | 2 |  |
|  |  | Kumpi buli lunaku | 3 |  |
| 8 | Okutambula oba okwogera empola ennyo n’abantu abalala ne bakiraba. Oba okuba nga toterera mukifo kimu, okwata kano ne kali era nga otambula tambula nnyo odda eno odda eri okusinga bulijjo. | Nedda | 0 |  |
|  |  | Enaku ezimu | 1 |  |
|  |  | Enaku ezisinga obungi | 2 |  |
|  |  | Kumpi buli lunaku | 3 |  |
| 9 | Okulowoza nti wandibadde bulungi nsinga offudde oba okulowooza okwetusaako obulumi (obuvune) mungeri yonna. | Nedda | 0 |  |
|  |  | Enakku ezimu | 1 |  |
|  |  | Enakku ezisinga obungi | 2 |  |
|  |  | Kumpi buli lunaku | 3 |  |
|  |  |  |  |  |
|  |  |  |  |  |
